# Supplementary material for: Activation of stably silenced genes by recruitment of a synthetic de-methylating module
Source: Nat Commun. 2022 Sep 23;13:5582. doi: 10.1038/s41467-022-33181-4 (PMC9508233; doi:10.1038/s41467-022-33181-4)
Supplement: Supplementary file 1 — Supplementary Information [file 41467_2022_33181_MOESM1_ESM.pdf]

Supplementary Information

Activation of stably silenced genes by recruitment of a synthetic de-methylating module

Supplementary Figures

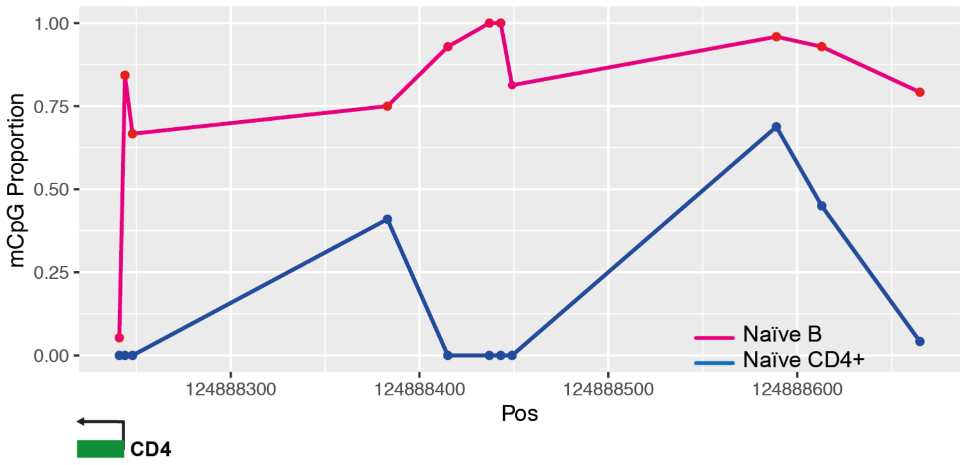

Supplementary Figure 1. ***Cd4*** **promoters are differentially methylated between B and T cells.** DNA methylation profiles of naïve B and CD4+ T cells at the *Cd4* promoter, retrieved from GSE94674[13], plotted as population proportion of methylated cytosine in each CpG dinucleotide motif.

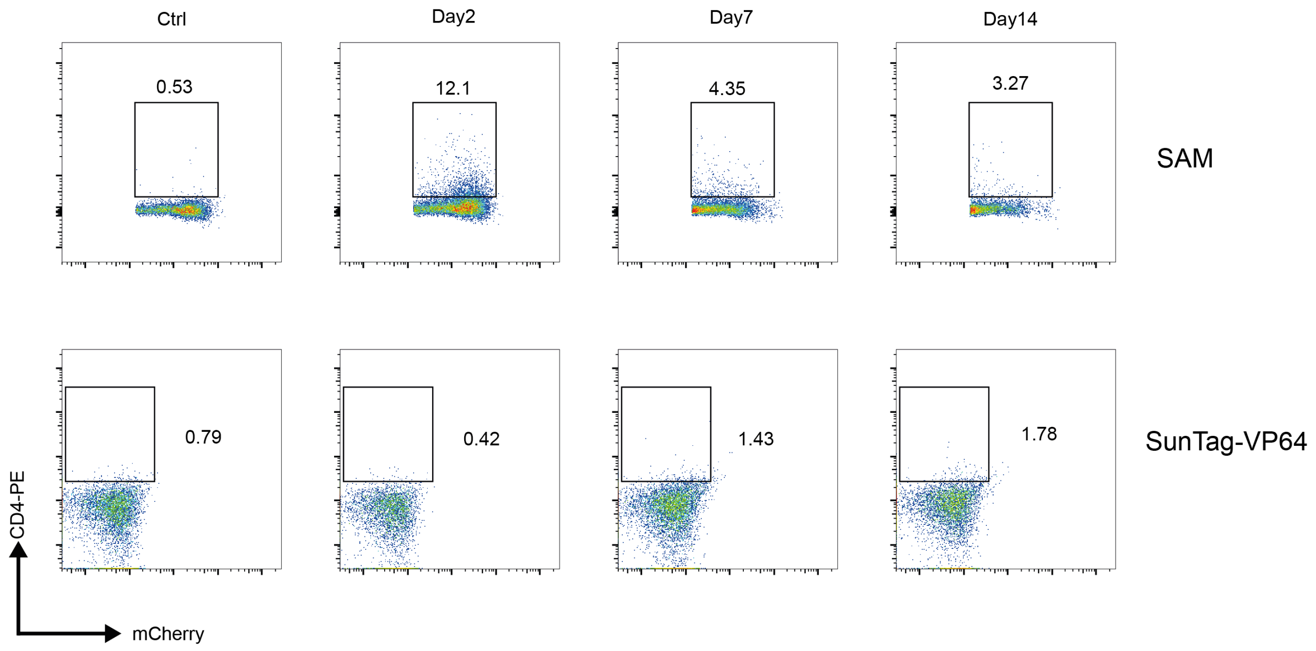

Supplementary Figure 2. SAM and SunTag-VP64 are unable to robustly upregulate CD4 in B cells. Representative flow cytometry plots showing CD4 surface expression in A20 with SAM or SunTag-VP64 constructs and transduced with *Cd4*-targeting sgRNA on the indicated day post-sgRNA-transduction. Positive gates for each time point were set against the negative population of cells transduced with control sgRNA on the same day.

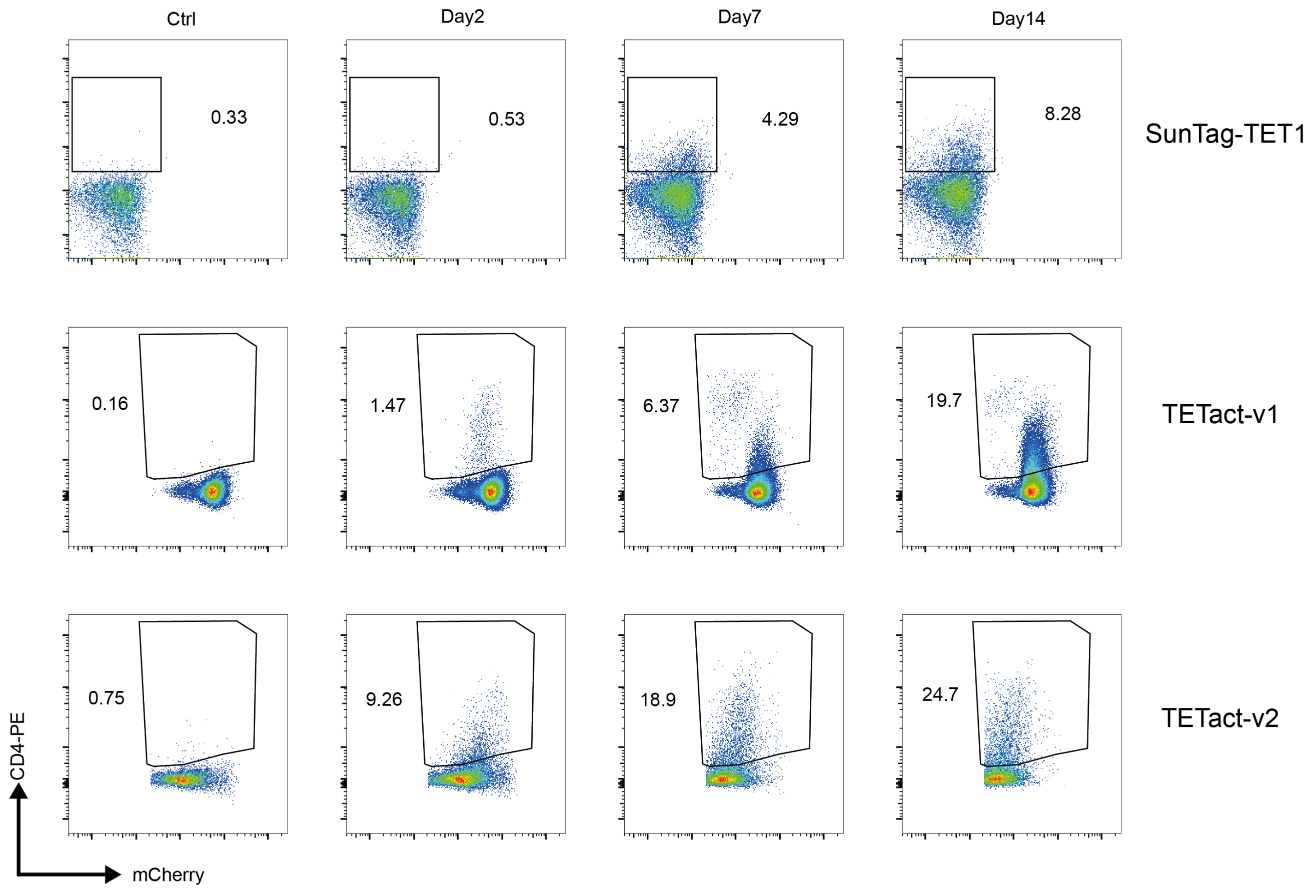

Supplementary Figure 3. Activation of CD4 using other TET1-associated systems. Representative flow cytometry plots showing CD4 surface expression in A20 with SunTag-TET1, TETact-v1 or -v2 constructs and transduced with *Cd4*-targeting sgRNA on the indicated day post-sgRNA-transduction. Positive gates for each time point were set against the negative population of cells transduced with control sgRNA on the same day.

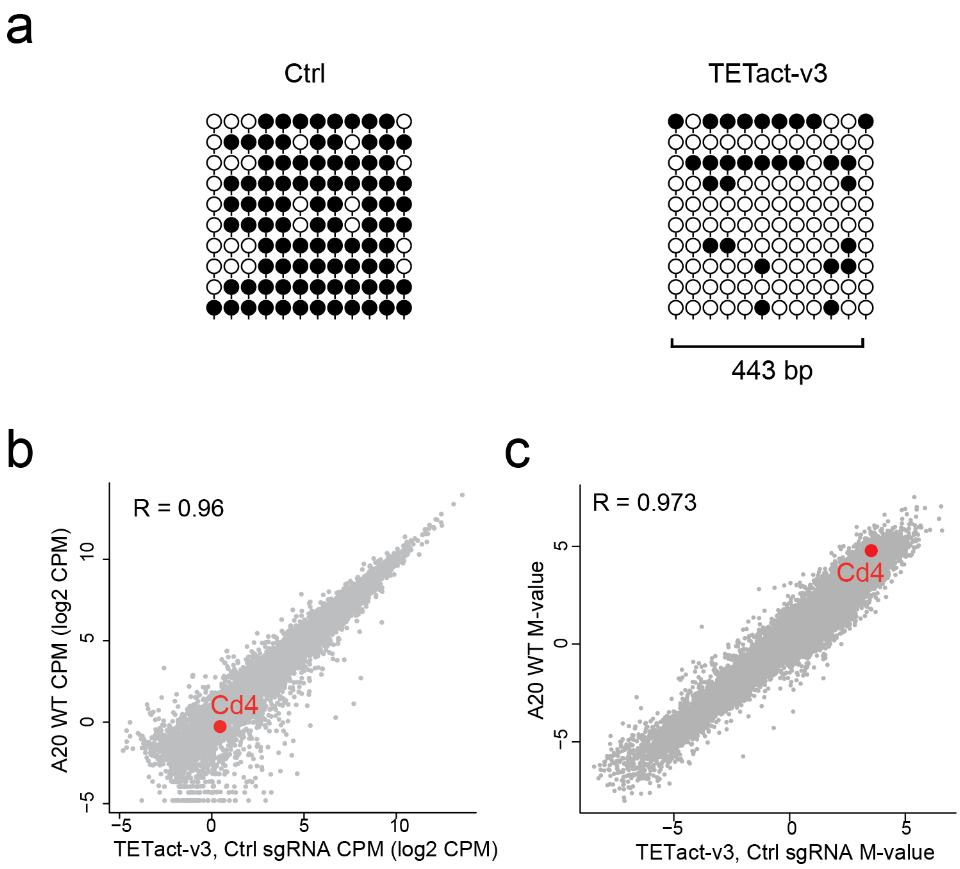

Supplementary Figure 4. Bisulphite sequencing, RNA-seq and EM-seq off-targets validation. (**a**) Bisulphite sequencing of the *Cd4* promoter in A20-TETact-v3 cells transduced with either control or *Cd4* promoter-targeting sgRNA. Open lollipops represent nonmethylated CpG dinucleotides whereas closed lollipops represent methylated dinucleotides. Each row represents an individual clone. Ten clones were analysed in each group. (**b**) Gene expression (log_2CPM) or (**c**) DNA methylation level (M-value) of promoters in cells of WT or versus gene expression in cells transduced with non-targeting control sgRNA. Transduced cells were assayed on day 7 post-sgRNA-transduction. R denotes the Pearson's correlation co-efficient which was calculated for log-transformed values on all genes/promoters that survived filtering except *Cd4*. The average of two biological replicates within a group is shown.

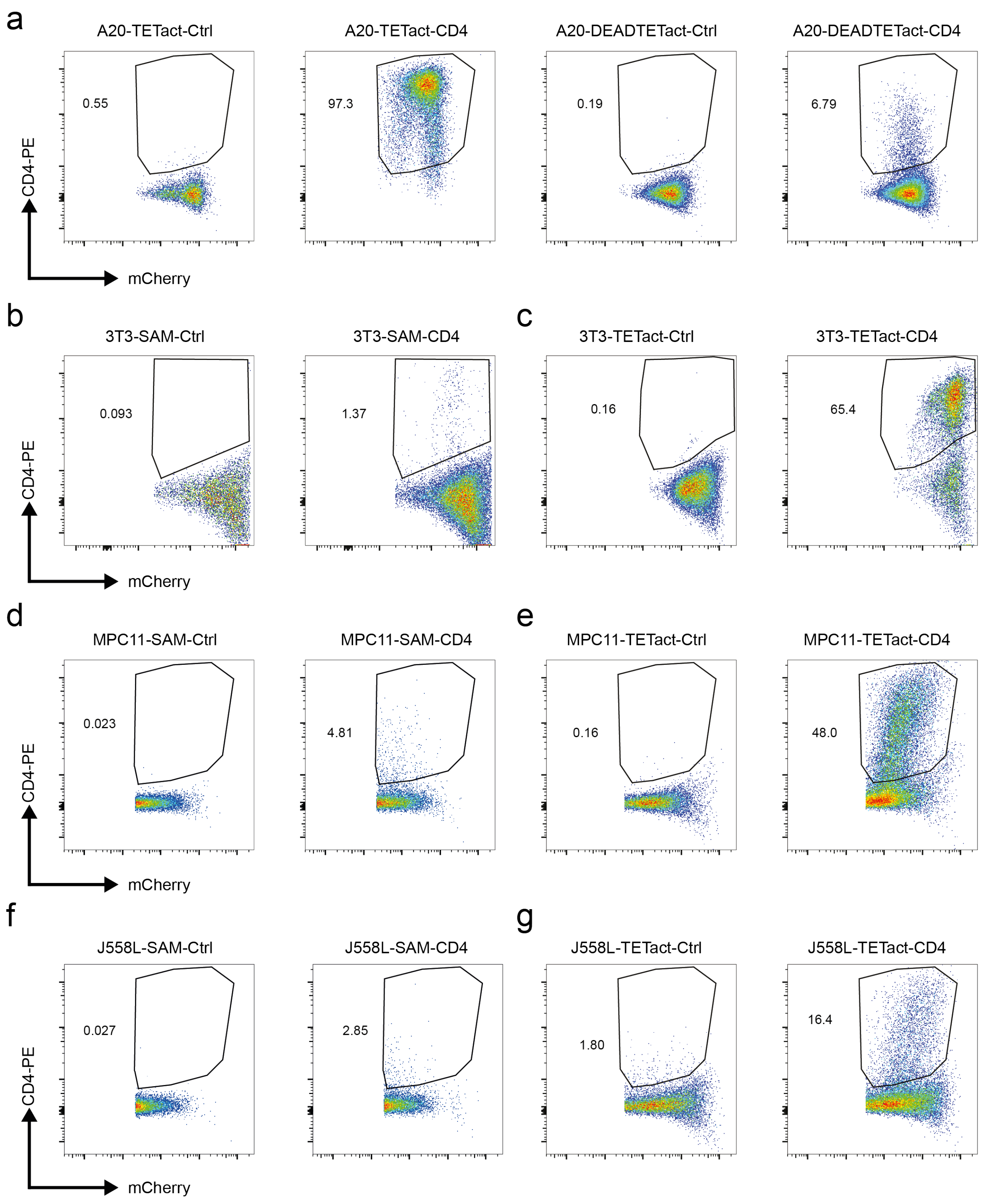
a
A20-TETact-Ctrl
A20-TETact-CD4
A20-DEADTETact-Ctrl
A20-DEADTETact-CD4
0.55
97.3
0.19
6.79
CD4-PE
mCherry
b
3T3-SAM-Ctrl
3T3-SAM-CD4
c
3T3-TETact-Ctrl
3T3-TETact-CD4
0.093
1.37
0.16
65.4
CD4-PE
mCherry
d
MPC11-SAM-Ctrl
MPC11-SAM-CD4
e
MPC11-TETact-Ctrl
MPC11-TETact-CD4
0.023
4.81
0.16
48.0
CD4-PE
mCherry
f
J558L-SAM-Ctrl
J558L-SAM-CD4
g
J558L-TETact-Ctrl
J558L-TETact-CD4
0.027
2.85
1.80
16.4
CD4-PE
mCherry

Supplementary Figure 5. TETact requires catalytic activity of TET1 to mediate gene induction, and is capable of activating genes in multiple cell lines. Representative flow cytometry plots showing CD4 surface expression in (**a**) A20-TETact cells and cells bearing the catalytically dead TET1CD (DEADTETact); (**b**) 3T3 with SAM activators; (**c**) 3T3 with TETact activators; (**d**) MPC11 cells with SAM activators; (**e**) MPC11 cells with TETact activators; (**f**) J558L with SAM activators and (**g**) J558L cells with TETact activators. All cells were either transduced with the control or *Cd4*-targeting sgRNA as indicated and assayed on 7 days post-sgRNA-transduction. Positive gates were drawn based on the negative population of cells transduced with control sgRNA in the same experiment.

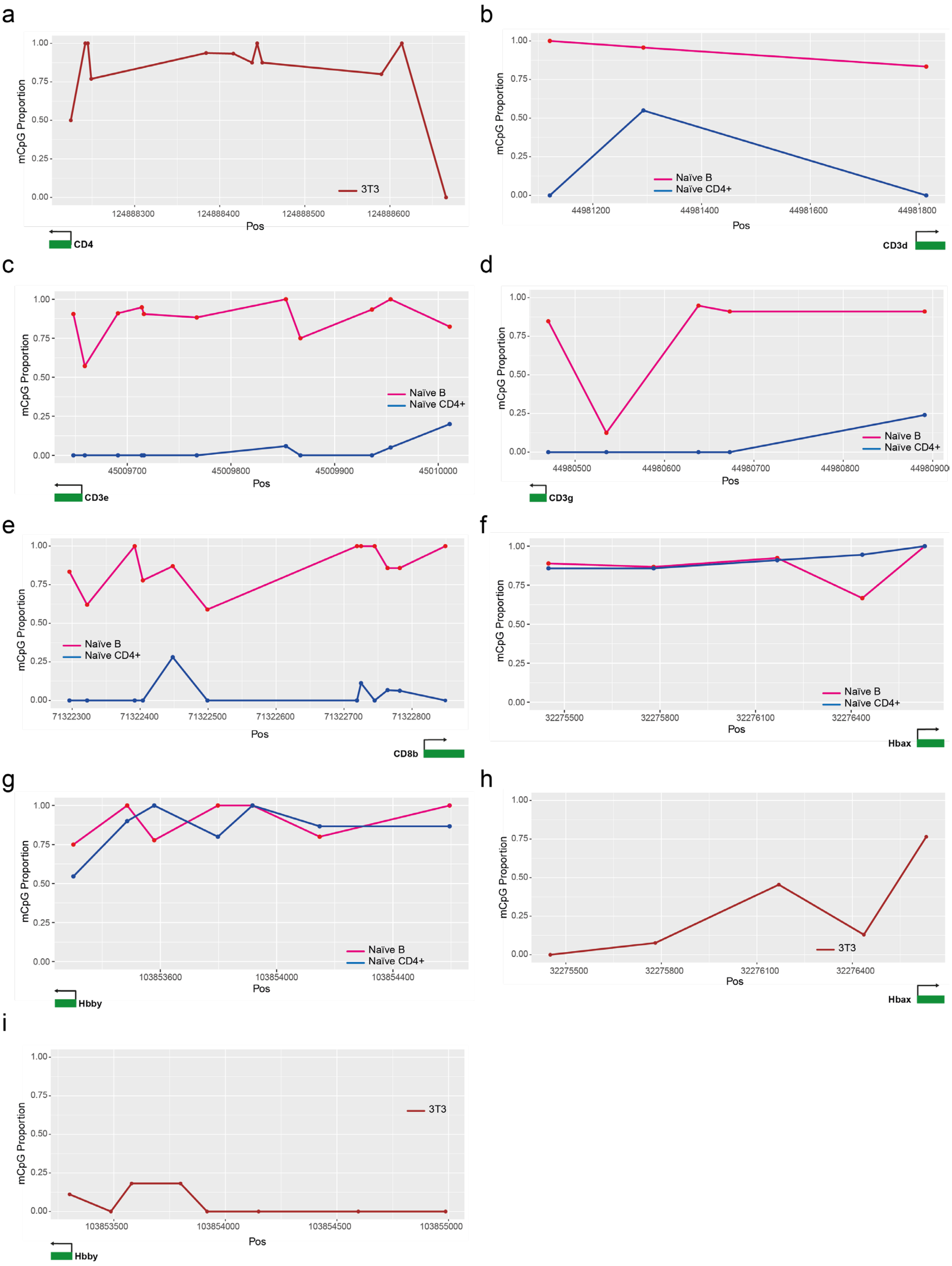
a
mCpG Proportion
Pos
3T3
CD4
b
Naïve B
Naïve CD4+
CD3d
c
CD3e
d
CD3g
e
CD8b
f
Hbax
g
Hbby
h
i

Supplementary Figure 6. DNA methylation status of target gene promoters. DNA methylation profiles of (**a**) 3T3 cells at the *Cd4* promoter; naïve B and CD4+ T cells at the (**b**) *Cd3d*; (**c**) *Cd3e*; (**d**) *Cd3g*; (**e**) Cd8b; (**f**) *Hba-x* and (**g**) *Hbb-y* promoters, and 3T3 cells at the (**h**) *Hba-x* and (**i**) *Hbb-y* promoters. WGBS data for 3T3 cells were retrieved from GSE162138[17] whereas that for B and T cells were retrieved from GSE94674[13]. Profiles were plotted as population proportion of methylated cytosine in each CpG dinucleotide motif.

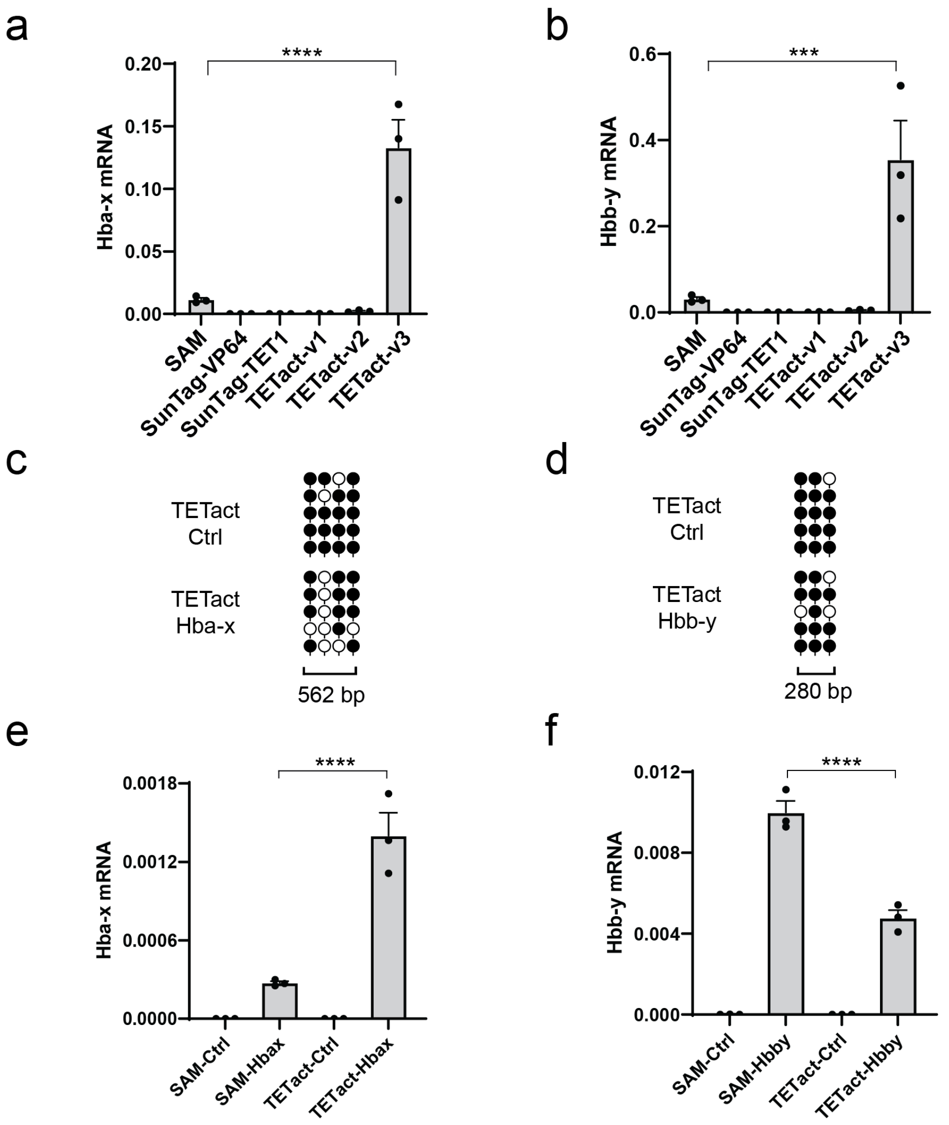

Supplementary Figure 7. Activation of embryonic globin genes. (**a**, **b**) Expression of *Hba-x* and *Hbb-y* in A20 cells transduced with the promoter-targeting sgRNA in different systems as indicated. $P < 0.0001$ (*Hba-x*), $P = 0.0002$ (*Hbb-y*) from one-way ANOVA with Dunnett's *post hoc* test compared to SAM. (**c**, **d**) Bisulphite sequencing of *Hba-x* and *Hbb-y* promoters for A20-TETact cells transduced with either control or the corresponding sgRNAs. Open lollipops indicate non-methylated CpG dinucleotides whereas closed lollipops represent methylated CpG motifs. Each row represents an analysed clone. Five clones were analysed in each group. (**e**, **f**) Expression of *Hba-x* and *Hbb-y* in 3T3 cells transduced with either control or the promoter-targeting sgRNA in different systems as indicated. Expression level is relative to *Actb* as $2^{-\Delta Ct}$. $P < 0.0001$ from one-way ANOVA with Tukey's *post hoc* test. Data are shown as mean ± s.e.m. from 3 independent transductions. ***$P < 0.001$, ****$P < 0.0001$. Source data are provided as a Source Data file.

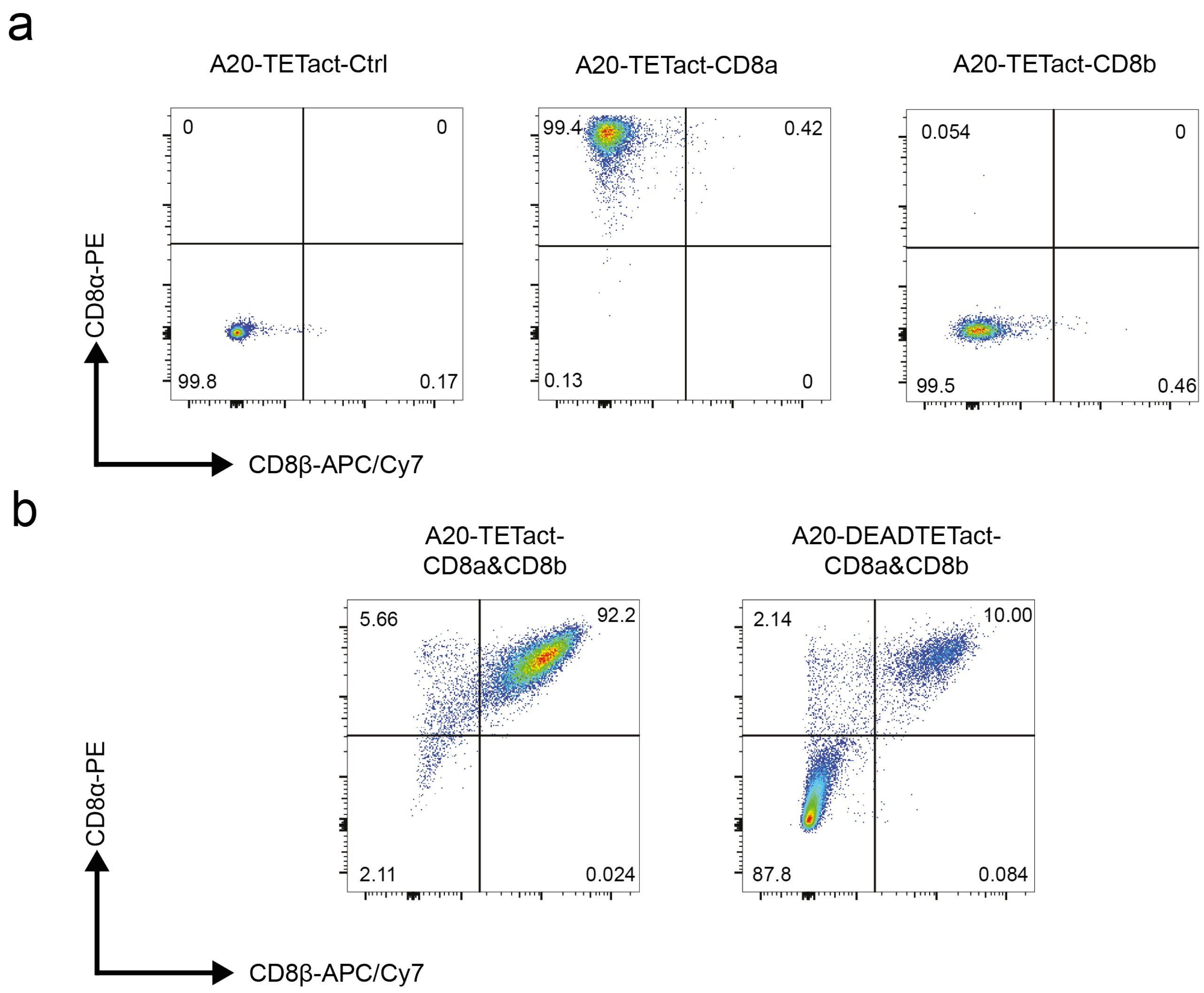

Supplementary Figure 8. Simultaneous activation of CD8α and CD8β in A20 cell lines. (**a**) Representative flow cytometry plots showing CD8α and CD8β surface expression in A20-TETact cells transduced with either *Cd8a*- or *Cd8b*-targeting sgRNA. (**b**) Representative flow cytometry plots showing CD8α and CD8β surface expression in A20-TETact and A20-DEADTETact cells transduced with vector co-expressing *Cd8a*- and *Cd8b*-targeting sgRNAs. Cells were assayed on day 7 post-sgRNA-transduction. Gates were drawn based on the negative population of cells transduced with control sgRNA in the same experiment.

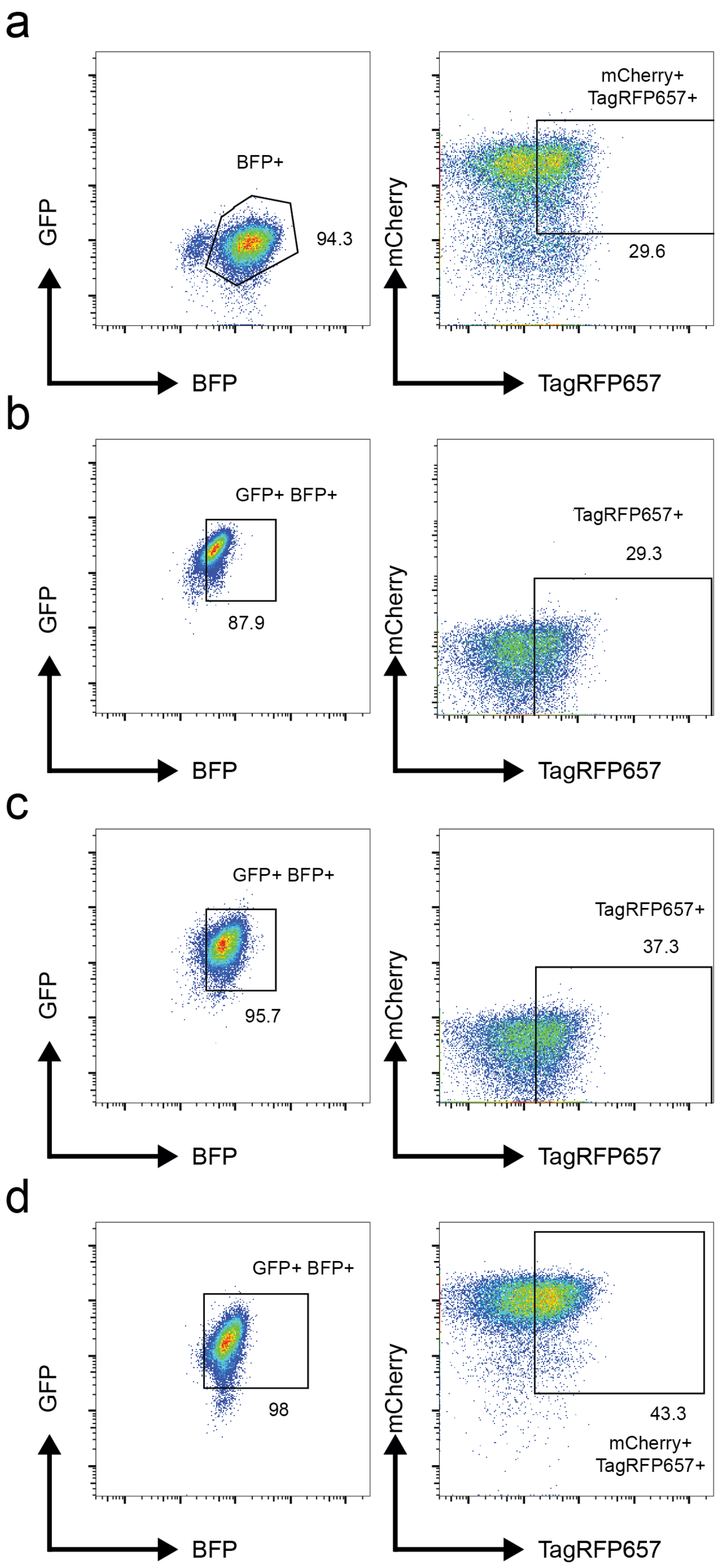

Supplementary Figure 9. Gating strategies for flow cytometry. Representative flow cytometry plots showing gating strategies for analysing or isolating (**a**) SAM, (**b**) SunTag-VP64, (**c**) SunTag-TET1, (**d**) TETact v1-3 and DEADTETact.

Supplementary Table 1: Primers for plasmid construction

Plasmids	Targets	Sequence
dCas9-5xGCN4-P2A-BFP	5xGCN4	AATAGGATCCAACGGTCCGACTGACGC
		AATAGCGGCCGCGACCCGGACCCTGAGCCCCCAGAC
MCP-p65-hsf1-T2A-mCherry	T2A-mCherry	ATTAGCTAGCGGCAGTGGAGAGGGCAGAGGAAGTCTGCTAACATG
		ATTAGAATTCTTACTTGTACAGCTCGTCCATGCCG
gRNA-MS2x2-TagRFP657	BbsI cassette	ACCGGTGTCTTCGAGGCTTACAGGACGAAGACCC
		AAACGGGTCTTCGTCCTGTAAGCCTCGAAGACAC
	Adding XbaI	TGTGGAAAGGACGAAACACCGGTGTC
		TATAGAATTCAGTATTTCTAGACTCTCGAGGTCGACATTTCAAAAAAAGCACCGACTCG
MCP-VP64-p65-hsf1-mCherry	VP64-p65-hsf1	AAGGTGGCGGCCGCTGGATCCGATGCTTTAGACGATTTTGACTTAGATATGCTT
		CGCTGAAGCCGCTGCCGCTGCCAGAG
		CAGCGGCAGCGGCTTCAGCGTGG
		GCCCTCTCCACTGCCGCTAGCGGAGACAGTGGG
MCP-VPR-mCherry	VPR	TAATTAACCTGCTATGGATCCAGTGATGCTTTAGACGATTTTGACTTAGATATG
		ATTATAACCTGCTATGCTAGCAAACAGAGATGTGTCGAAGATGGAC
scFv-GCN4-sfGFP-deadTET1CD	sfGFP-deadTET1CD	GGAGGCGGCGGAAGCTTGGATC
		TGTGAATGGCCCTGTAGGGATGAG
		CTCATCCCTACAGGGCCATTCACA
		AAGGGTCGACTCTAGAGTCGCG
MCP-p65-hsf1-BFP	P2A-BFP	ATTAGCTAGCGGCAGTGGAGCTACTAACTTCAGCCTGCTGAAGCAG
		ATTAGAATTCTTAATTAAGCTTGTGCCCCAGTTTGC
Multiplex sgRNAs	F1	GAGTGAAGACTTCTAGATTAGTGAACGGATCTCGACG
	R1	CTCAGAAGACAATTTTGACTCTCGAGGTCGACATTTC
	F2	GAGTGAAGACTTAAAAATTAGTGAACGGATCTCGACG
	R2	CTCAGAAGACAAAATTGACTCTCGAGGTCGACATTTC

Supplementary Table 2: gRNA target sequences

Targets		gRNA sequence
Dreg1	+38	AACATTCCCATGCGATGCTC
	-25	AAAGCTTACATGGACCAACC
	-32	ACATGGACCAACCAGGAGTG
Cd4		AGCCTGGTTAGGTCAACGTG
Hba-x		CAACAATGGGAATTAGGGCT
Hbb-y		ATGACCTGGCTCCACCCATG
Cd3e		TCCTCAGTTAACCAAGGCGG
Cd3d		TGACAGTCTTACACCATCAA
Cd3g		CAGAGCATCAACACCCCTGC
Cd8a		GGTGGTTGACACTCTTTGGT
Cd8b		GACTTTTCAAAGAGCCCACC

Supplementary Table 3: Bisulphite sequencing PCR primers

Cd4	F	TAAATGAAAAGATAGGAAGTTTGG
	R	CACTATATCTTTAACTACCACA
Dreg1	F	GAGGAAAATTTTGATTTTTTGATTTTTT
	R	TCACTCCTAATTAATCCATATAAACTTT
Hba-x	F	TGTATTTTATTGTATATTGGTTGGTATG
	R	TAATACCTAAACTTACCTCTCTAAAATC
Hbb-y	F	TATTTTTTGAAGTTATTGGTTAGTTTGA
	R	AATATTAACATTTCTATATCCACAACAC
Cd3e	F	GAGAGATTTATTATTTTTGAAGAAGGTA
	R	ACAAAAACAAAAACTATATAACTCTCTC
Cd3d	F	GTAGTTTGTATTTGTTTGTGTTTTATTT
	R	AAAAAACAACAATCAATATCAAACTAAC
Cd3g	F	TTGGTTTTATTTAGTATGTATTGAGTTG
	R	TAAAAATCTAACTTTCCACACTACTATA
Cd8b	F	TGGGTTTTATTTGTTAGTTTTATATTGA
	R	TAACTAAATCTAAAATCTATCACTTCCT

Supplementary Table 4: qRT-PCR primers used in study

Targets		gRNA sequence
Actb	F	GACTCATCGTACTCCTGCTTG
	R	GATTACTGCTCTGGCTCCTAG
	Probe	CTGGCCTCACTGTCCACCTTCC
Dreg1	F	CTTTGCTCTTCACTCTGGACT
	R	CTGTGTCCCATGCTTCTCTG
	Probe	TCAAAGCCCCAGATTCCTTCATCTCC
Hba-x	F	CCATTGGCACTGAGACTCTAG
	R	CTCTTAACCGCATCCCCTAC
Hbb-y	F	TGCTGACTGCTTTTGGAGAG
	R	ACCAGCACATTACCCAAGAG
Cd3e	F	TGGAGCAAGAATAGGAAGGC
	R	CATAGTCTGGGTTGGGAACAG
Cd3d	F	AACTCTGCTCCTGGCTTTG
	R	GCTGTACTGGGTATCTTCACG
Cd3g	F	GTATATCTCATTGCGGGACAGG
	R	TCCTCAGTTGGTTTCCTTGG
Cd8b	F	TGGCCGTCTACTTTTACTGTG
	R	GGCGCTGATCATTTGTGAAAC
