# Supplementary material for: Activation of stably silenced genes by recruitment of a synthetic de-methylating module
Source: Nat Commun. 2022 Sep 23;13:5582. doi: 10.1038/s41467-022-33181-4 (PMC9508233; doi:10.1038/s41467-022-33181-4)
Supplement: Supplementary file 2 — Reporting Summary [file 41467_2022_33181_MOESM2_ESM.pdf]

nature portfolio

Corresponding author(s): Wing Fuk Chan and Rhys Allan

Last updated by author(s): Sep 6, 2022

Reporting Summary

Nature Portfolio wishes to improve the reproducibility of the work that we publish. This form provides structure for consistency and transparency in reporting. For further information on Nature Portfolio policies, see our Editorial Policies and the Editorial Policy Checklist.

Statistics

For all statistical analyses, confirm that the following items are present in the figure legend, table legend, main text, or Methods section.

n/a	Confirmed	
[ ]	[x]	The exact sample size (*n*) for each experimental group/condition, given as a discrete number and unit of measurement
[ ]	[x]	A statement on whether measurements were taken from distinct samples or whether the same sample was measured repeatedly
[ ]	[x]	The statistical test(s) used AND whether they are one- or two-sided<br>*Only common tests should be described solely by name; describe more complex techniques in the Methods section.*
[x]	[ ]	A description of all covariates tested
[x]	[ ]	A description of any assumptions or corrections, such as tests of normality and adjustment for multiple comparisons
[ ]	[x]	A full description of the statistical parameters including central tendency (e.g. means) or other basic estimates (e.g. regression coefficient) AND variation (e.g. standard deviation) or associated estimates of uncertainty (e.g. confidence intervals)
[ ]	[x]	For null hypothesis testing, the test statistic (e.g. *F*, *t*, *r*) with confidence intervals, effect sizes, degrees of freedom and *P* value noted<br>*Give P values as exact values whenever suitable.*
[x]	[ ]	For Bayesian analysis, information on the choice of priors and Markov chain Monte Carlo settings
[x]	[ ]	For hierarchical and complex designs, identification of the appropriate level for tests and full reporting of outcomes
[ ]	[x]	Estimates of effect sizes (e.g. Cohen's *d*, Pearson's *r*), indicating how they were calculated

Our web collection on statistics for biologists contains articles on many of the points above.

Software and code

Policy information about availability of computer code

Data collection	BD FACSAria Fusion, BD FACSAria III, BD FACSymphony A3, BD LSRFortessa, BIORAD CFX384, QuantStudio 6 Flex, NextSeq 2000.
Data analysis	FlowJo 10.4.1, GraphPad Prism 9, Bisulfite Primer Seeker (https://www.zymoresearch.com/pages/bisulfite-primer-seeker), IDT online design tool (https://www.idtdna.com/SciTools), SnapGene 5.1.0, RStudio/ggplot v3.3.5, Rsubread v2.8.1 (Liao et al., 2013), edgeR v3.36.0 and v3.38.1 (McCarthy, Chen, & Smyth, 2012), limma v3.50.0 (Ritchie et al., 2015), TrimGalore v0.6.7 (Krueger, 2012), bismark v0.20.0 (Krueger & Andrews, 2011), bsseq v1.32.0 (Hansen et al., 2012), DMRcate v2.10.0 (Peters et al., 2015).

For manuscripts utilizing custom algorithms or software that are central to the research but not yet described in published literature, software must be made available to editors and reviewers. We strongly encourage code deposition in a community repository (e.g. GitHub). See the Nature Portfolio guidelines for submitting code & software for further information.

Data

Policy information about availability of data

All manuscripts must include a data availability statement. This statement should provide the following information, where applicable:

- Accession codes, unique identifiers, or web links for publicly available datasets
- A description of any restrictions on data availability
- For clinical datasets or third party data, please ensure that the statement adheres to our policy

RNA-seq data generated in this study is accessible in GSE203162.
Whole genome EM-seq data generated in this study is accessible in GSE211754.
Public dataset for whole genome bisulphite sequencing for B and T cells is accessible in GSE94674 under the SuperSeries GSE94676 from Kazachenka et al., 2018.

Public dataset for whole genome bisulphite sequencing for 3T3 cells is accessible in GSE162138 from Sapozhnikov & Szyf, 2021.

Field-specific reporting

Please select the one below that is the best fit for your research. If you are not sure, read the appropriate sections before making your selection.

[x] Life sciences [ ] Behavioural & social sciences [ ] Ecological, evolutionary & environmental sciences

For a reference copy of the document with all sections, see nature.com/documents/nr-reporting-summary-flat.pdf

Life sciences study design

All studies must disclose on these points even when the disclosure is negative.

Sample size

No statistical method was used to pre-determine sample size but sample sizes were chosen based on researchers' experience as well as experimental replicates in various published literature such that they enable statistical analyses. All CRISPR-related data (qRT-PCR, flow cytometry) was generated from 3 independent experiments, with sample size in line with other published studies.
5-10 clones were analysed for Bisulphite sequencing, the number of analysed clones is in line with various published literature, and is sufficient to reveal the pattern and difference due to the treatment.
Sequencing experiments were performed in duplicate. From previous experience, RNA-seq and EM-seq experiments are reproducible with duplicate libraries showing minimal variation. These were sufficient to determine differences between samples.

Data exclusions

No exclusion

Replication

All CRISPR-related data (qRT-PCR and flow cytometry) was generated from 3 independent experiments, all attempts gave similar results. 5-10 individual clones were analysed for bisulphite sequencing. RNA-seq and EM-seq have been replicated twice and it gives similar results.

Randomization

Randomization is not relevant to working with wildtype cell lines as cell lines are clonal and share identical genotype. However, once stable transfectants are generated through lentiviral transduction, only cells as bulk population with random lentiviral integration (without obtaining single-cell clones) were analysed in all subsequent studies. Samples were only grouped according to the treatment and otherwise randomised.

Blinding

FACS technicians were blinded to the sample identities during sorting the samples. Sequencing platform technicians were blinded to the nature of the experiments. Bioinformaticians were blinded to the nature of the samples prior to the analyses.

Reporting for specific materials, systems and methods

We require information from authors about some types of materials, experimental systems and methods used in many studies. Here, indicate whether each material, system or method listed is relevant to your study. If you are not sure if a list item applies to your research, read the appropriate section before selecting a response.

Materials & experimental systems

n/a	Involved in the study	
[ ]	[x]	Antibodies
[ ]	[x]	Eukaryotic cell lines
[x]	[ ]	Palaeontology and archaeology
[x]	[ ]	Animals and other organisms
[x]	[ ]	Human research participants
[x]	[ ]	Clinical data
[x]	[ ]	Dual use research of concern

Methods

n/a	Involved in the study	
[x]	[ ]	ChIP-seq
[ ]	[x]	Flow cytometry
[x]	[ ]	MRI-based neuroimaging

Antibodies

Antibodies used

CD4-PE, clone #GK1.5, WEHI in-house, used in dilution 1:800;
CD8a-PE, clone #53-6.7, BioLegend #100707, used in dilution 1:800;
CD8b-APC/Cy7, clone #YTS156.7.7, BioLegend #126619, used in dilution 1:600.

Validation

Antibody validations were performed by antibody suppliers, also the antibodies have been validated and titrated by using positive/negative control cells from C57BL/6 mouse in many other experiments before the current studies, which demonstrate they stain the cell types that express the surface markers detected by these antibodies.
Validation data for commercial antibodies are available on vendor website.
CD8a-PE, clone #53-6.7:
https://www.biolegend.com/en-us/products/pe-anti-mouse-cd8a-antibody-155
CD8b-APC/Cy7, clone #YTS156.7.7, BioLegend #126619

https://www.biolegend.com/en-us/products/apc-cyanine7-anti-mouse-cd8b-antibody-10021

Eukaryotic cell lines

Policy information about cell lines

Cell line source(s)	HEK293T (ATCC, CRL-3216)<br>A20 (ATCC, TIB-208)<br>3T3 (ATCC, CRL-1658)<br>MPC11 (ATCC, CCL-167)<br>J558L (ECACC, 88032902)
Authentication	HEK293T, A20, 3T3, MPC11 and J558L were authenticated by the morphology according to ATCC or ECACC data or other published results. No additional authentication was conducted. HEK293T cells only used for lentiviral production.
Mycoplasma contamination	HEK293T, A20, 3T3, MPC11 and J558L cells were not tested for mycoplasma contamination. HEK293T cells only used for lentiviral production.
Commonly misidentified lines<br>(See ICLAC register)	No commonly misidentified lines

Flow Cytometry

Plots

Confirm that:

- [x] The axis labels state the marker and fluorochrome used (e.g. CD4-FITC).
- [x] The axis scales are clearly visible. Include numbers along axes only for bottom left plot of group (a 'group' is an analysis of identical markers).
- [x] All plots are contour plots with outliers or pseudocolor plots.
- [x] A numerical value for number of cells or percentage (with statistics) is provided.

Methodology

Sample preparation	Cell line was used.
Instrument	Flow cytometric analyses were perfomed on BD FACSymphony A3 or LSRFortessa. Cell sorting were performed on BD FACS Aria III or BD FACSAria Fusion.
Software	FlowJo v10.4.1
Cell population abundance	Purity of post-sort population was checked and always exceeded 98%.
Gating strategy	All cells were gated on FSC-A/SSC-A to exclude dead cells, followed by doublet exclusion by FSC-H/FSC-A. All cells were first gated on GFP and BFP, then gated on mCherry and TagRFP657. For SAM-transduced cells, cells were sorted as BFP+ mCherry + TagRFP657+; SunTag-VP64 or SunTag-TET1 cells were sorted as BFP+ GFP+ TagRFP657+; TETact v1-v3 cells were sorted as BFP+ GFP+ mCherry+ TagRFP657+ population. For surface marker studies, from the previous gating, cells were further gated on either CD4-PE vs mCherry, CD8a-PE vs CD8b-APC/Cy7 or CD4-PE vs CD8b-APC/Cy7.

- [x] Tick this box to confirm that a figure exemplifying the gating strategy is provided in the Supplementary Information.
