# Supplementary material for: Activation of stably silenced genes by recruitment of a synthetic de-methylating module
Source: Nat Commun. 2022 Sep 23;13:5582. doi: 10.1038/s41467-022-33181-4 (PMC9508233; doi:10.1038/s41467-022-33181-4)
Supplement: Supplementary file 4 — Description of Additional Supplementary Files [file 41467_2022_33181_MOESM4_ESM.pdf]

Title: Supplementary Data 1.
Description: Differential gene expression analysis results for i) A20 with sgRNA activating CD4 vs A20 with non-targeting control sgRNA and ii) A20 with non-targeting control sgRNA vs wild-type A20. Genes are ranked by B-statistic (log-odds).

Title: Supplementary Data 2.
Description: Gene promoter differential methylation analysis results for the three pair-wise comparisons. Gene promoters are ranked by p-values.
